# A novel stereoscopic projection display system for CT images of fractures

**DOI:** 10.3892/etm.2013.1044

**Published:** 2013-04-03

**Authors:** XIUJUAN LIU, HONG JIANG, YUEDONG LANG, HONGBO WANG, NA SUN

**Affiliations:** 1 Department of CT Room, 1st Affiliated Hospital of Harbin Medical University, Harbin, Heilongjiang 150001;; 2 Department of CT Room, Heilongjiang Province Hospital Nangang Branch, Harbin, Heilongjiang 150001;; 3 Beijing Aerospace Control Instrument Research Institute, Beijing 100854;; 4 School of Mechatronics, Harbin Institute of Technology, Harbin, Heilongjiang 150001, P.R. China

**Keywords:** projection, stereo display, computed tomography image, fracture

## Abstract

The present study proposed a novel projection display system based on a virtual reality enhancement environment. The proposed system displays stereoscopic images of fractures and enhances the computed tomography (CT) images. The diagnosis and treatment of fractures primarily depend on the post-processing of CT images. However, two-dimensional (2D) images do not show overlapping structures in fractures since they are displayed without visual depth and these structures are too small to be simultaneously observed by a group of clinicians. Stereoscopic displays may solve this problem and allow clinicians to obtain more information from CT images. Hardware with which to generate stereoscopic images was designed. This system utilized the conventional equipment found in meeting rooms. The off-axis algorithm was adopted to convert the CT images into stereo image pairs, which were used as the input for a stereo generator. The final stereoscopic images were displayed using a projection system. Several CT fracture images were imported into the system for comparison with traditional 2D CT images. The results showed that the proposed system aids clinicians in group discussions by producing large stereoscopic images. The results demonstrated that the enhanced stereoscopic CT images generated by the system appear clearer and smoother, such that the sizes, displacement and shapes of bone fragments are easier to assess. Certain fractures that were previously not visible on 2D CT images due to vision overlap became vividly evident in the stereo images. The proposed projection display system efficiently, economically and accurately displayed three-dimensional (3D) CT images. The system may help clinicians improve the diagnosis and treatment of fractures.

## Introduction

The stereoscopic display of fractures is required by clinicians since the diagnosis and treatment of fractures are highly dependent on computed tomography (CT) post-processing images for manual reduction. Shaded volume rendering (SVR) is commonly used in hospitals as an effective and convenient method for reconstructing stereo CT images in CT workstations. Three-dimensional (3D) models may be reconstructed from sequential slices via SVR and shown on two-dimensional (2D) screens or medical films; the structures are made translucent to allow the clinicians to observe the interior of the bone (1,2). However, these 3D images are too small to be simultaneously observed by a group of clinicians. In addition, the visual data regarding the mutual location, size and shape of bone fragments in fractures often overlap or are not visible since these images are displayed without visual depth. Therefore, the evaluation of complex anatomical regions requires considerable experience from clinicians and involves several potential difficulties (3).

The 3D structures and shapes from volumetric data are difficult to perceive in translucent volumes without depth cues (4), whereas a feeling of depth during monitoring may provide more information (5). To overcome these difficulties, Kniss *et al* (6) combined volume rendering with a virtual reality display. Shen *et al* (7) described a real-time medical visualization system for analyzing volumetric data in various virtual environments. In the system provided by Ji *et al* (8), stereo viewing was achieved using a head-mounted display (HMD). Zhang *et al* (9) proposed a method for stereo image generation from video data using phase correlation technology. Without 3D information, respective motion parameters for the formation of a stereo pair for the left and right eyes were computed, from which the stereo images were generated (8). Zinger *et al* (10) presented another approach for the autostereoscopic display of medical images and developed an efficient algorithm for view interpolation and rendering, which was based on texture-depth data representation. In addition to the virtual reality methods, mixed-reality methods have been applied for medical image displays. Ferrari *et al* (11) generated augmented images to provide users with a stereoscopic view. Sielhorst (12) studied the medical application of depth perception using translucent head-mounted displays (HMDs). These studies were conducted in laboratories, but medical applications of a technology have more practical requirements. To provide more accessible CT or MRI medical images to patients, Okuyama *et al* (13) reconstructed stereoscopic CT and MRI images and displayed them on a notebook personal computer by adopting binocular-type stereoscopy. These studies enabled simple and low-cost autostereoscopy with a small-scale system (14). However, this desktop system does not fulfill the requirements of cooperative work among doctors during diagnosis.

The present study presents a novel method of projection display based on a virtual reality enhancement environment system (VREES). The proposed system stereoscopically displays fractures with the advantages of clear images and low operating costs. This system converted the post-processing 2D images from CT workstations into large 3D images, which may be simultaneously displayed to several clinicians for discussion and consultation. Therefore, clinicians are able to obtain more information from the 3D images than from the 2D images and treat patients with fractures with greater efficacy.

## Materials and methods

### Hardware and procedure

**Figure 1:**
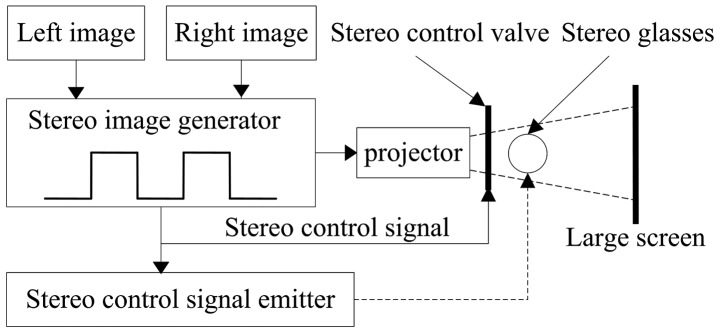
Three dimensional (3D) projection system based on 3D image generator.

Active stereo projection was adopted as the display method due to its generality, feasibility and cost. A stereo generating device was designed to replace the advanced graphics cards and to decrease the system costs. In this system, the image resources were obtained by the off-axis algorithm and synthesized as a time-sequenced image pair by a 3D image generator. The image pair were projected onto the screen and the control signals were transmitted to a 3D control valve to drive the shutter of the 3D glasses. The modules of the system are shown in Fig. 1.

One advantage of the proposed system is that only low-level drivers are required for stereo display, instead of professional graphics cards. The key problem was the creation of the image pair for the left and right eyes in real-time. The medical images were automatically saved by the CT workstation as a sequence with different viewpoints that may be used as input images. The off-axis algorithm transformed the source images into stereo pairs in the software module of the proposed system. Therefore, stereo projection display was achieved with a standard personal computer and a consumer-level stereo projector.

The following equipment was used in the VREES to cost-effectively and efficiently display 3D images; a computer, a large display screen, a projector, a 3D-image generator, 3D glasses and 3D control emitters. The equipment may be purchased at reasonable prices, with the exception of the 3D image generator, which was manufactured.

The 3D image generator in our proposed system was composed of five parts: an input-and-output interface, a power interface, a pulse-generating unit, an image-processing unit and a 3D-driving unit. Three tasks had to be completed by the system while the generator was operational. The first task was to obtain the ordered images according to the pulse signals from the pulse generating unit and to feed these images into the 3D-image generator according to the same time sequence. The second task was to generate 3D signals according to the time rules of the vertical sync signal from the pulse generating unit and to alternately translate these signals into the next unit. The third task was to produce the driving 3D control signal, which is used to synchronize the opening and closing of the shutters of the 3D glasses with the vertical sync signal.

**Figure 2:**
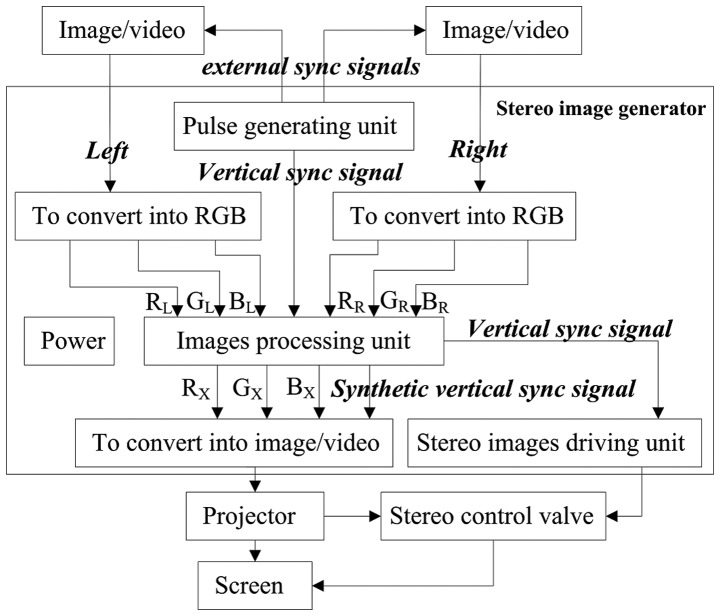
Control circuit principle of three dimensional (3D) image generator.

The control circuit of the 3D image generator is shown in Fig. 2. Images or videos were used as sources of the 3D signals, which were sent to the generator through the left and right input interfaces. These left and right signals were converted to the RGB format by the input interface. Two signals were then synchronously selected and controlled with a specific time sequence in the pulse generating unit.

The workflow of the generator in the proposed system had five steps. i) The 3D image generator first obtained the input image or video signals and estimated whether the refresh frequencies of the two-way signals were synchronous. If the refresh frequencies were not synchronous, the ongoing procedure was terminated and the graphics card started the refresh synchronization mode. ii) When the refresh frequencies were synchronous, the video synchronization mode opened the switch control of the two-way image signals. iii) The image processing unit then created a 3D image signal for the high-brightness projector. iv) Once the generator finished creating the appropriate 3D image signal, the video graphics array (VGA) interface exported this signal to a high-brightness projector, whereas a special interface exported the 3D synchronous control signal to the infrared emitter of the stereo glasses. Otherwise, the procedure returned to Step 1. v) After the signals were exported, the procedure exited from the loop. The generator was then stopped.

The selection and control of the two processed signals was performed in real time, such that these were synchronous with the input signals. The left image signal (RL-GL-BL) and the right image signal (RR-GR-BR) were alternately and continuously obtained in a fixed order until all the 3D signals (RX-GX-BX) were generated.

### Software algorithm

Based on the principle of stereo projection, human eyes are able to receive stereo images due to the disparity between the two eyes and between the independent images. In the present study, two conditions ensured the generation of a stereo display. The first aspect was the accuracy of the left and right images for the required distances as well as the disparity angles of the two eyes for stereo imaging. The second aspect was the absolute separation of the left and right eyes for the independent images, and the image consistency in terms of color and illumination. To obtain the images for the left and right eyes, the projection model for binocular projection was established in the system. Similarly, the left and right cameras were calibrated for the 3D construction of the two images that were observed from different viewpoints by the two cameras.

Stereo image pairs with specific constraints were generated by the left and right images from the two cameras in the proposed system. To simplify the 3D construction process, rapid image generation was achieved by rotating the angles and by translating the distances of the two cameras. The toe-in, on-axis and off-axis algorithms are commonly used methods for image generation. In the toe-in algorithm, a different view is simulated by simply rotating the cameras. However, vertical errors occur from the image distortion that is caused by views in non-parallel directions. The on-axis algorithm creates the observation area by translating the positions of two cameras, but focusing problems of the left and right eyes may be encountered. The off-axis algorithm creates the fixed camera position and computes the projection matrix for the left and right eyes by translating the range of viewpoints.

**Figure 3:**
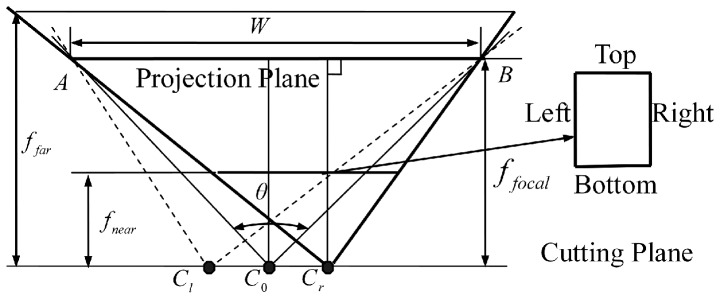
Parameter calculation of stereo visual mode.

Between these three algorithms, the off-axis algorithm was able to render stereo images with optimal visual effects. The visual cones of the two eyes are not symmetrical, and the directions of the viewpoints are parallel in this algorithm. However, the resulting vertical disparity may cause discomfort. Thus, the present study used the off-axis algorithm to generate the stereo images, as shown in Fig. 3.

Based on the off-axis algorithm, C_l_ and C_r_ were set as the left and right camera centers, respectively, whereas the representation of the projection plane boundary was deduced by referring to 3D Stereo Rendering Using OpenGL (and GLUT) (15). The formulae to calculate right camera parameters were:

$$\begin{array}{c} l_{\mathrm{left}}=-k_{w}.f_{\mathrm{near}}\cdot \text{tan}\left(\frac{\theta }{2}\right)+\frac{f_{\mathrm{near}}}{f_{\mathrm{focal}}}\cdot e\\ l_{\mathrm{right}}=k_{w}\cdot f_{\mathrm{near}}\cdot \text{tan}\left(\frac{\theta }{2}\right)+\frac{f_{\mathrm{near}}}{f_{\mathrm{focal}}}\cdot e\\ l_{\mathrm{top}}=f_{\mathrm{near}}\cdot \text{tan}\left(\frac{\theta }{2}\right)\\ l_{\mathrm{bottom}}=-f_{\mathrm{near}}\cdot \text{tan}\left(\frac{\theta }{2}\right) \end{array}$$

where *f_near_* is the distance of the near plane of camera, *f_far_* is the distance of the far plane of camera, *θ* is the view angle of the camera, *f_focal_* is the focal length of the camera, *l_left_*, *l_right_*, *l_top_* and *l_bottom_* are the edges length of clipping plane, *e* is half the distance between the left and right cameras and *k_w_* is the ratio of the camera width and height:

$$k_{w}=\frac{l_{\mathrm{right}}-l_{\mathrm{left}}}{l_{\mathrm{top}}-l_{\mathrm{bottom}}}$$

The projection matrix of the right camera (*M_rproj_*) was then represented as:

$$M_{\mathrm{rproj}}=\left[\begin{array}{cccc} \frac{2f_{\mathrm{near}}}{l_{\mathrm{right}}-l_{\mathrm{left}}} & 0 & \frac{l_{\mathrm{right}}+l_{\mathrm{left}}}{l_{\mathrm{right}}-l_{\mathrm{left}}} & 0\\ 0 & \frac{2f_{\mathrm{near}}}{l_{\mathrm{top}}-l_{\mathrm{bottom}}} & \frac{l_{\mathrm{top}}+l_{\mathrm{bottom}}}{l_{\mathrm{top}}-l_{\mathrm{bottom}}} & 0\\ 0 & 0 & \frac{f_{\mathrm{far}}+f_{\mathrm{near}}}{f_{\mathrm{near}}-f_{\mathrm{far}}} & \frac{2f_{\mathrm{far}}}{f_{\mathrm{near}}-f_{\mathrm{far}}}\\ 0 & 0 & -1 & 0 \end{array}\right]$$

The projection matrix of the left camera (*M_lproj_*) was obtained in a similar manner Therefore, the stereo image pairs were obtained using projection matrixes *M_lproj_* and *M_rproj_*. The stereoscopic video was synthesized from images paired by the distributor. The switching of the signals was controlled in the cycles of the corresponding left and right channel signals. The left signal was first transmitted in one cycle and the right signal was transmitted in the next cycle. This switching continued with the Rx, Gx, Bx, horizontal (Hx) and vertical (Vx) signals in five channels. The vertical refresh rate of the synthesized stereo signal remained constant since the (Vx) was included in both channels. The switching of the two signals during their synchronous transmission was controlled according to the vertical scanning frequency of the refreshing signals. The stability of the output image was ensured by the constant vertical scanning frequency. Finally, the stereo video signals were sent to the projector for real-time display. The driving signal of the synchronous stereo display was sent to the infrared stereo signal emitter, where it controlled the shutters of the liquid crystal glasses for the users to observe stereo images with depth information.

## Results

### Large-scale

**Figure 4:**
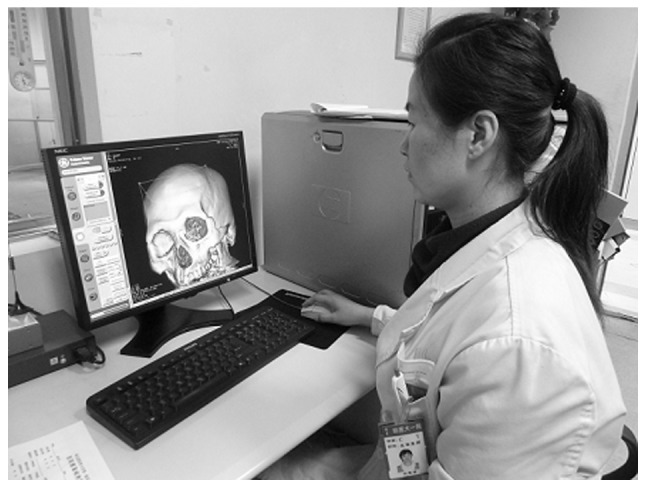
Use of a VRS by a single doctor. VRS, virtual reality system.

**Figure 5:**
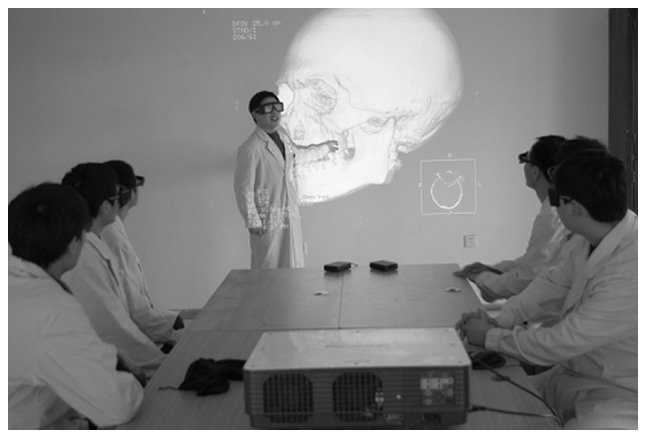
Application of the VREES for discussion and consultation. VREES, virtual reality enhancement environment system.

Compared with conventional virtual reality system (VRS) displays, the 3D visualization of fractures using VREES was observed to be a more convenient and efficient method for groups of clinicians to diagnose and discuss fractures. As shown in Fig. 4, a single clinician operated the computer for observing the CT image on the VRS desktop. The resulting 2D images were too small to be simultaneously observed and discussed by several clinicians. By contrast, a maximum of seven clinicians with 3D glasses observed the 3D images clearly on a large scale when using the VREES. Each clinician received identical and complete information from the 3D images (Fig. 5).

### 3D display of fractures with local defects

**Figure 6:**
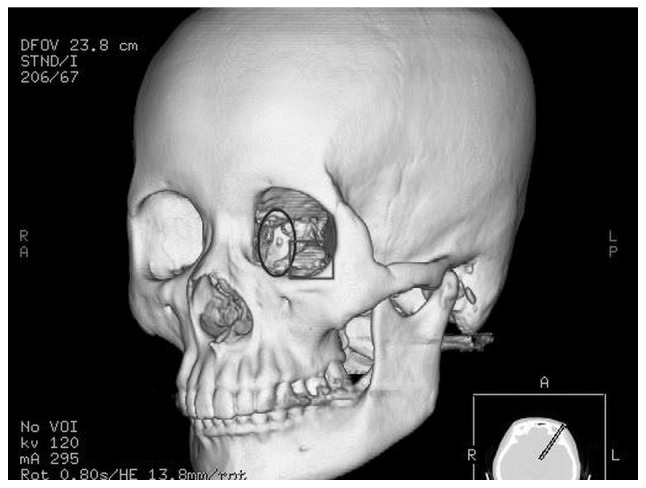
Medial orbital walls in the right eye with local defects (circle) and inferior orbital wall fracture (rectangle).

To illustrate the advantages of the 3D images of fractures generated by the VREES, several applications of the proposed system on fracture images were performed. Certain parts of the lamina cribrosa in the medial orbital wall of the eye were too thin to be selected as bone volume elements within the limits of the CT values in SVR. Therefore, these parts were not shown in the CT images. The complete medial orbital wall with its local defects was not observed by the ophthalmologists. As shown in Fig. 6 (circle), the images of the medial orbital walls with local defects in the eye overlapped and were not clear. These walls were close to each other. Therefore, the 2D images lacked visual depth. The shape of the orbital walls, the fracture itself, and the placement of the fracture along the medial orbital wall in the eye were difficult for clinicians to observe and diagnose. Consequently, the information remained hidden in the 2D images. By contrast, when the visual depth of the image was reconstructed in the VREES, the two medial orbital walls of the eye were displayed distinctly. The fracture line and the sclerotin with local defects as well as the size and displacement of the bone fragments were clearly visible. With the aid of 3D images in the VREES, ophthalmologists were able to calculate the quantity and shape of the artificial bone pieces. These pieces were used to improve the accuracy of filling in local defects of the sclerotin and the positioning of titanium plates on fractures.

The inferior orbital wall, which is opposite the upper maxillary sinus wall, had an uneven thickness in the obtained images. The CT images showed that the thinness of this wall was similar to that of the medial orbital wall in the eye. In addition, the orbital fossa formed tetrahedron and the 2D images overlapped without visual depth. Thus, the sclerotin of the inferior orbital wall was not clearly visible in the 2D images, as shown in Fig. 6 (rectangle). By contrast, the sclerotin of the inferior orbital wall and the fracture were clearly displayed with the reconstruction of visual depth in the VREES.

### 3D display of fractures without displacement

**Figure 7:**
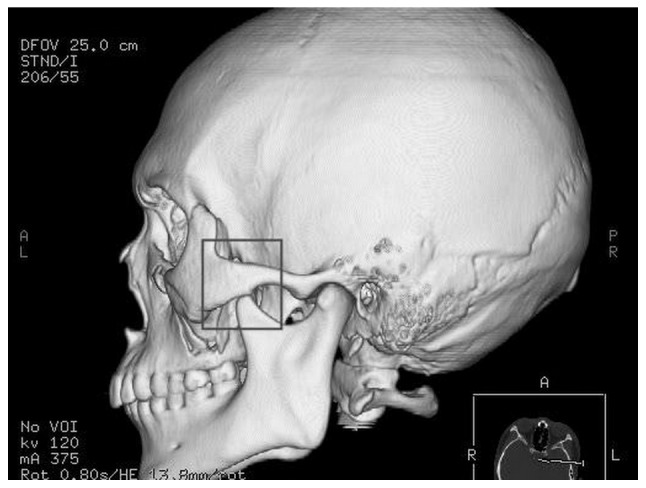
Fracture without displacement.

Different types of human tissue were shown in SVR to have varying diaphaneity. When one type of tissue was observed through a different tissue, the 2D images of these two tissues obscured each other. Consequently, fractures without displacement or those with a displacement of <2 mm were generally unclear, as shown in Fig. 7 where the fracture is not visible. However, fracture lines that were not visible in the 2D images were clearly and vividly displayed in the VREES since the 3D images showed the stereo view of the bone to the orthopedists. The orthopedists were able to observe the fracture from various directions, such that all signs of the fracture were detectable. This ability to observe fractures from any direction is of great significance to orthopedists for the diagnosis and evaluation of fractures, including those that are extremely small.

### 3D display of fractures with displacement, rotation and distance of fracture ends

**Figure 8:**
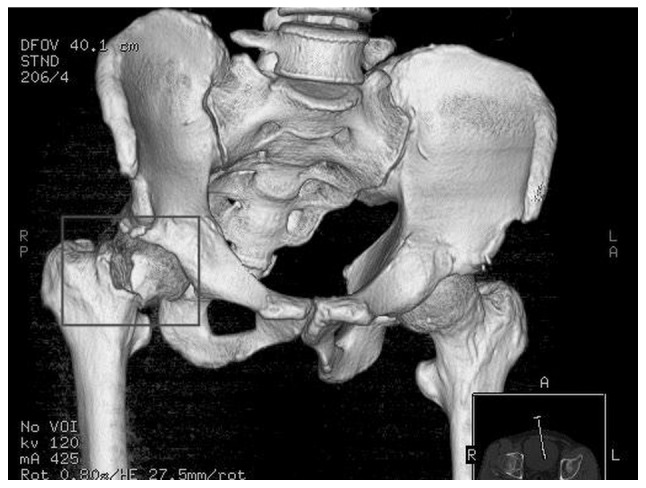
Displacement, rotation and distance of fractured ends.

The displacement of a fracture in the intra-articular, front and back regions, as well as the size of the bone fragments and the fracture site, were all presented in the single, unclear 2D image that was generated in SVR without visual depth, as shown in Fig. 8. To obtain more information on a fracture, doctors were able to rotate the 2D images on-screen with SVR. However, the doctors were then required to mentally reconstruct a 3D model of the fracture site. Therefore, the information obtained by one doctor may differ from that obtained by others, which may make it difficult for the doctors to agree. By contrast, the 3D fracture information was clearly shown in the VREES. The doctors obtained identical information regarding the articular cavity, thereby enabling them to easily come to an agreement and reasonably select the most suitable surgical approach. Furthermore, the doctors were able to effectively use the fixation method for accurate fracture reduction.

## Discussion

Stereo CT images of fractures are commonly displayed on a plate screen using the volume element reconstruction method (16). The stereo projection display that is featured in the VREES produced images in a virtual reality system that were able to help clinicians diagnose various conditions, plan treatment options and monitor changes over time as well as predict and display final treatment results (6,17). Applications of the proposed system showed that the visual depth was reconstructed in the 3D images that were produced in the VREES. Stereo CT images were clearly displayed by the proposed system, including the size, shape and displacement of bone fragments as well as the front and back displacements of the fracture. Certain fractures were not visible in the 2D CT images due to image overlap. However, these fractures were vividly displayed in the VREES. These results are consistent with those of previous virtual reality methods (9,10). The proposed system efficiently meets the requirements of clinicians for fracture observation, but also provides a large visual scope for orthopedists to conveniently discuss fractures in groups.

Numerous software systems are installed in CT machines to provide 3D images on flat screens. Clinicians generally observe 3D models on 2D display devices. If clinicians wish to see planar 3D images in their respective departments, the special software that is required for CT machines is unavailable due to prohibitive pricing.

Stereo projection display systems consist of a computer, a large screen or white wall, a projector, a 3D image generator, 3D glasses and 3D control emitters, the majority of which are conventional devices in meeting rooms (18). HMDs are coupled with 3D perspective-rendering algorithms in mixed-reality systems (19,20). By contrast, stereo projection display systems use fewer and less expensive devices. Thus, stereo projection display systems are relatively economical, thereby allowing clinicians in underdeveloped areas to clearly observe the fractures of their patients as easily as their counterparts in developed areas.

A stereo projection display system is just one method for clinicians to observe 3D CT images. Clinicians usually focus on the accuracy and convenience of the systems being used. However, the source images of the proposed system are derived from CT post-processing images, which are the original CT images without modification. Thus, the information obtained by clinicians from the 3D images in the proposed system is similar to that from the original CT post-processing images. Therefore, the accuracy of the proposed system is undisputed. However, clinicians are often uncomfortable with wearing 3D glasses. If 3D images may be observed without 3D glasses, clinicians may find the system more comfortable and convenient. Therefore, future studies on a system that does not require 3D glasses are required.
